# Babies Beginning to Walk

**Published:** 1889-11

**Authors:** 


### Babies Beginning to Walk.

The age at which babies begin the wide based waddle, which they believe to represent walking, varies some. It is not an unheard of thing for them to struggle to their feet as early as eight months old. Nor is it unusual to prolong the creeping stage far beyond the twelve months age. When a baby insists on walking before ten or eleven months, it is a sign that it is a strong and active child. When it delays until eighteen months the reverse is indicated; there is surely some weakness that prevents it. Notwithstanding these facts, it is a mistake to "teach" a child to walk. Whatever the Darwinian theory about how we came to assume the upright position, our children are "to the manner born," and will take it as soon as nature lets them. One thing only is a legitimate help, and that is putting the child on the floor as soon as possible for it to roll and kick for itself. The floor should be thoroughly protected from draughts, and a warm rug or puff placed under the baby. Then give him his fling, in company with a few rubber toys, and it will soon be found that the rug is too small for him. He becomes more enterprising, and manages to get off by rolling, "hitching," and scrambling.

This sort of exercise is perfectly natural to a healthy child, and helps to develop the soft legs and feet, and to digest and assimilate food. Besides that, the moral effect is to give the child a sort of self-reliance. Those who "hold the baby" too long cheat him of just so much of his little experience. Let him scramble and wallow, regardless of mopping up the dust or getting grimy; or, if you cannot bear to see him dirty, make him a "creeper" of dark stuff, to be slipped off and on at pleasure. Soon he reaches up, as all growing things do, or should, and finds something above him to grasp; then pulls himself up and stands on his feet. The next stage is walking, by holding on to chairs, the wall, skirts of dresses, and other inanimate friends. After that, in time, he walks. Babies come to all this as natural as they cry when hungry, but should never be forced or coaxed for the amusement of their elders.—*Herald of Health.*

---

It is necessary from time to time to sound a note of warning with respect to the possible dangers of cocaine. A correspondent sends us the following cutting from a local paper. *Narrow Escape.*--Mr. F. A. Dixey, of Wadham College, had a narrow escape from death last Thursday week. A visit to a dentist is not often associated with danger, but it appears that Mr. Dixey, who had to undergo a somewhat painful operation to his teeth, received an injection in his gums of hydrochlorate of cocaine, and from constitutional or other causes almost succumbed to the effect of the anodyne. We understand that two doctors were called in, and it was long before Mr. Dixey was brought to consciousness. He was removed to the Acland Home on Saturday, and is now making satisfactory progress.

One of the "sweet graduates" in a neighboring town, says the Sumter County, Florida, *Times*, read an essay on physiology, in which she said : "The human body is divided into three parts—the head, the chist and the stomick. The head contains the eyes and brains, if any. The chist contains the lungs and a piece of the liver. The stomick is devoted to the bowels, of which there are five, a, e, i, o, u, and sometimes w and y.—*Western Reporter.*

---

We often cry out about dental advertisements, forgetting that almost every calling must necessasily have its black sheep, and that the medical profession—to which we look up as our model—has to contend with the same difficulties both here and abroad. Dental advertisers are not without ingenuity in this country, but they are quite out of the field as compared with some "tall" work done on the other side of the water, as will appear from the following paragraph which recently appeared in the *Illustrated Medical News:*—There is too much tendency in the present day to resort to personal advertisement, and to bring one's name before the public in non-medical newspapers. But, happily, we have never yet in this country reached the standard of perfection which has been arrived at in America. In an American paper we find the following heading : "A Curious Way to Advertise an *Obstetrician.*"

"Flamin.—Saturday, the 9th inst., at 8:15 a. m., to the wife of D. W. Flamin, of College Hill, a ten-pound boy. Thanks to Dr. Wallingford, of Cincinnati.

"Gallion.—June 5th, to Mrs. Nona Gallion, of Liberty Street, a nine-pound girl. Thanks to Dr. Wallingford."

When reading such a notice one naturally feels inclined to look at another side of the question, and ask whether no thanks are due Messrs. Flamin and Gallion as well as to Dr. Wallingford. But we can hardly suppose that Mesdames Flamin and Gallion would have permitted such a notice to be inserted in the paper in question were it not with the consent of Dr. Wallingford.
—*Dental Record, Lond.*

